# Transcriptional Reprogramming at Genome-Scale of *Lactobacillus plantarum* WCFS1 in Response to Olive Oil Challenge

**DOI:** 10.3389/fmicb.2017.00244

**Published:** 2017-02-17

**Authors:** María Esteban-Torres, Inés Reverón, Laura Plaza-Vinuesa, Blanca de las Rivas, Rosario Muñoz, Félix López de Felipe

**Affiliations:** Laboratorio de Biotecnología Bacteriana, Instituto de Ciencia y Tecnología de los Alimentos y Nutrición – Consejo Superior de Investigaciones CientificasMadrid, Spain

**Keywords:** transcriptomics, *Lactobacillus plantarum*, olive oil, stringent response, fatty acid biosynthesis

## Abstract

Dietary fats may exert selective pressures on *Lactobacillus* species, however, knowledge on the mechanisms of adaptation to fat stress in these organisms is still fragmentary. This study was undertaken to gain insight into the mechanisms of adaptation of *Lactobacillus plantarum* WCFS1 to olive oil challenge by whole genome transcriptional profiling using DNA microarrays. A set of 230 genes were differentially expressed by *L. plantarum* WCFS1 to respond to this vegetable oil. This response involved elements typical of the stringent response, as indicated by the induction of genes involved in stress-related pathways and downregulation of genes related to processes associated with rapid growth. A set of genes involved in the transport and metabolism of compatible solutes were downregulated, indicating that this organism does not require osmoprotective mechanisms in presence of olive oil. The fatty acid biosynthetic pathway was thoroughly downregulated at the transcriptional level, which coincided with a diminished expression of genes controlled by this pathway in other organisms and that are required for the respiratory function, pyruvate dehydrogenase activity, RNA processing and cell size setting. Finally, a set of genes involved in host-cell signaling by *L. plantarum* were differentially regulated indicating that olive oil can influence the expression of metabolic traits involved in the crosstalk between this bacterium and the host.

## Introduction

Recent studies have shown that the type of dietary fat has a direct impact in the composition of the gut microbiota (de Wit et al., 2012; Patterson et al., 2014) however, the mechanisms underlying these changes are poorly understood.

Olive oil, the main fat of the Mediterranean diet, is considered premium oil from a nutritional viewpoint (Estruch et al., 2013). This vegetable oil but not butter, shapes the host microbial ecosystem by increasing the population of the *Lactobacillaceae* family (Hidalgo et al., 2014). Gaining insight into the mechanisms by which olive oil exert selective pressures on lactobacilli could be a step forward to better understand the transformation changes in the gut microbiota induced by this oil. In addition, knowledge on how olive oil stress the metabolic traits of lactobacilli can be of utility to better understand the uneven impact of these organisms in relation to metabolic diseases linked to fat intake (Karlsson et al., 2011; Drissi et al., 2014; Li et al., 2014; Toral et al., 2014; Heeney et al., 2016), more in view that *Lactobacillus* species constitute a major population in the small intestine (Kleerebezem and Vaughan, 2009) where all fats are adsorbed (Krajmalnik-Brown et al., 2012).

Information on the mechanisms of adaptation of lactobacilli to olive oil is also of interest to apply this vegetable oil as vehicle for selected microorganisms that may contribute to a better management of gut microbial ecosystem and host health. Insights into the mechanisms by which olive oil exert selective pressures on lactobacilli may be of special interest for olive oil attributes, as the activity of olive microbiota during the oil extraction process (from which *Lactobacillus* species are part) could be a critical point for virgin olive oil quality (Vichi et al., 2011). In addition, this knowledge will expand the catalog of transcriptional datasets that has been recently launched to unravel the specific traits used by this organism to adapt to plants (Filannino et al., 2016).

Among lactobacilli, *Lactobacillus plantarum* is a suitable organism to study the mechanisms of adaptation to olive oil, because it successfully colonizes the olive and is part of the olive microbiota (Landete et al., 2010), occurs in our diets (Ahrné et al., 1998) and colonizes the gastrointestinal (GI)-tract from animals (de Vries et al., 2006). Therefore, to leverage information on the mechanisms of adaptation of *L. plantarum* to olive oil we examined the expression profile of the model strain *L. plantarum* WCFS1 in response to a challenge with this vegetable oil by transcriptomics.

Studying the effects of olive oil on the gene transcription in *L. plantarum* has also interest from a fundamental viewpoint. It is to note that *L. plantarum* feedback inhibits the endogenous biosynthesis of fatty acids upon the presence of exogenously added fatty acids (Sabaitis and Powell, 1976). At present, it is unclear whether this inhibition may be important for the biogenesis of *L. plantarum*, as it occurs in *Escherichia coli* (Yao et al., 2012) or in yeast mitochondria (Kursu et al., 2013) where the fatty acid biosynthesis (FAB) pathway dysfunction affects critical aspects of cell biogenesis. Considering that the biosynthesis of fatty acids is feedback inhibited in *L. plantarum* by fatty acids (Sabaitis and Powell, 1976), whole transcriptome microarray profiling in response to olive oil could decipher functions intersecting with the FAB pathway in this bacterium. In view of the above mentioned, the objective of this work is to provide insights on how virgin olive oil affects the expression profile of *L. plantarum* WCFS1 at genome scale.

## Materials and Methods

### Bacterial Strain, Culture Conditions, and Olive Oil

*Lactobacillus plantarum* WCFS1 was kindly provided by Dr. Michiel Kleerebezem (NIZO Food Research, The Netherlands) and grown in Man-Rogosa-Sharpe (MRS) broth (Difco Laboratories, Madrid, Spain) (de Man et al., 1960) at 30°C without shaking. This strain is a colony isolate of *L. plantarum* NCIMB 8826, which was isolated from human saliva and persists in the digestive tract of mice and humans better than other *Lactobacillus* sp. isolated from the human intestine (Vesa et al., 2003). Extra Virgin Olive Oil (EVOO) from the Picual variety harvested during the 2014–2015 season was obtained from a local producer (Jaén, south of Spain).

### RNA Extraction

Twelve paired independent *L. plantarum* WCFS1 batch cultures (50 mL each) were grown in MRS to an OD_600_ ≈ 0.8–0.9. Then, a culture of each pair (twelve cultures) was centrifuged at 9,000 × *g* for 5 min at 20°C and cells washed twice in 100 mM phosphate buffer pH 7.0. The washed cells were suspended in 0.5 ml of 100 mM phosphate buffer pH 7.0 and then induced with 0.5 ml of EVOO (final EVOO concentration 50%) by vortex-shaking during 10 min at room temperature. Washed cells (uninduced) were used as controls. These cells were suspended in 1 ml of 100 mM phosphate buffer pH 7.0 and subjected to vortex-shaking during 10 min at room temperature. The induced cells and their corresponding controls were cooled on ice for 7 min and centrifuged at 4°C. The pellet was mixed with 2 mL of quenching buffer (60% methanol, 66.7 mM HEPES, pH 6.5, –40°C). Following quenching, the cells were centrifuged at 9,000 × *g* for 10 min at –10°C and suspended in an extraction mixture (500 μL 1:4 chloroform-acid phenol, 30 μL of 10% SDS, 30 μL Na-acetate 3M pH 5.2, 400 μL Tris-EDTA buffer [10 mM Tris(hydroxymethyl)amino methane, 1 mM EDTA] pH 7.4, 15 mg of polyvinylpoly-pyrrolidone, and 500 mg of glass beads (ϕ, 75–150 μm). The cells were broken under frozen conditions in a FastPrep^TM^ Fp120 (SAVANT) using three treatments of 5000 rpm for 40 seconds and chilled 1 min between cycles. The suspension was then centrifuged at 4°C at 10,000 x *g* for 2 min. After two extractions with 500 μL of chloroform the supernatant containing the RNA was immediately frozen in liquid nitrogen, and stored at –80°C (Saulnier et al., 2007). NanoDrop ND1000 instrument was used for RNA quantification. The A_260_/A_280_ and A_260_/A_230_ ratios were measured to check RNA purity. Integrity and quality of RNA samples were determined by electrophoresis on agarose gels. Two treatments with DNase I (Ambion) were applied and the absence of genomic DNA was confirmed by PCR (Saulnier et al., 2007).

### Microarray: cDNA Synthesis, Purification, and Hybridization

Before first-strand cDNA synthesis, RNA integrity was evaluated using the Agilent 2100 Bioanalyzer (Agilent Technologies). Fluorescently labeled cDNA was obtained by using the SuperScript Indirect cDNA Labeling System (Invitrogen). After, the Cy3 and HyPer5 fluorescent dyes (Amersham Biosciences) were coupled to the aminoallyl-modified first-strand cDNA, and purification of probes was carried out with the CyScribe GFX Purification Kit. Labeling efficiency was assessed using a NanoDrop ND1000 spectrophotometer. Preparation of probes and hybridization at 65°C during 17 h was performed as described on the Two-Color Microarray Based Gene Expression Analysis Manual (Quick Amp Labeling) with Tecan HS Pro Hybridization (V. 5.7/Agilent Technologies). Slide *L. plantarum* WCFS1 8x15K microarray GE Agilent G2509F Oligo Microarrays (No. 026636) was custom designed and contains 60-mer probes that were taken at the gene expression omnibus database (GEO Accession No. GPL5874). The oligo-microarray contained an average of three probes per transcript.

Real-time quantitative RT-PCR assays (qRT-PCR). Real-time qRT-PCR was used to validate the microarray data. Amplification was carried out using a 7500 Fast System (Applied Biosystems). RNA was reverse transcribed using High Capacity cDNA Reverse Transcription Kits (Applied Biosystems). The specific primers used for the qRT-PCR assays are listed (see Additional file 1: Supplementary Table S1). The SYBR Green method was used and each assay was performed in triplicate using SYBR Green real-time PCR Master Mix (Applied Biosystems). Amplification was initiated at 95°C for 10 min, followed by 40 cycles of 95°C for 15 s and 60°C for 1 min. Control PCRs were included to confirm the absence of primer dimer formation (no-template control), and to verify that there was no DNA contamination (without RT enzyme negative control). All real-time PCR assays amplified a single product as determined by melting curve analysis and by electrophoresis. A standard curve was plotted with cycle threshold (*Ct*) values obtained from amplification of known quantities of cDNAs and used to determine the efficiency (*E*) as *E* = 10^-1/slope^. The expression levels of target genes were normalized. The Bestkeeper analysis (Pfaffl et al., 2004) was applied, and the geometric mean of the most stably expressed housekeeping genes (16S rRNA, *ldhD, gapB, dnaG*, and *gyrA*) was used as a normalization factor. The expression ratios measured by microarrays and by qRT-PCR assay were plotted, and the linear correlation coefficient was calculated (*y* = 1.2024x – 1.5843; *R*^2^= 0.89) (**Table 1**).

**Table 1:** RT-qPCR validation of nine differentially expressed genes according to microarray data.

Locus tag	Locus	Description	Expression ratios^a,b^	
			Microarray^c^	RT-qPCR^d^
*lp_1880*	–	Hypothetical protein	2.31	0.08
*lp_0997*	*cspC*	Cold sock protein CspC	1.8	1.01
*lp_0009*	*rpsF*	30S ribosomal protein S6	1.48	0.68
*lp_2755*	–	Membrane protein	1.34	0.45
*lp_2057*	*ldhD*	D-lactate dehydrogenase	1.14	0.38
*lp_2368*	*atpF*	H(+)-transporting two-sector ATPase, B subunit	–0.95	–3.04
*lp_1449*	–	Cell surface protein, CscB family	–1.27	–3.53
*lp_2035*	*aroE*	3-phosphoshikimate 1-carboxyvinyltransferase	–1.34	–3.71
*lp_0265*	*pts5ABC*	PTS system trehalose-specific transporter subunit IIBC	–2.16	–3.23
*lp_0007*	*gyrA*	DNA gyrase subunit A	–0.69	–2.93

*Linear regression fit analysis was applied and correlation coefficient used to validate the data (*y* = 1.002x – 1.584; *R*^2^ = 0.895). ^a^Genes ratios >1.5-fold changes (FC, either increase or decrease) were statistically significant (*p* < 0.05); except for *gyrA* gene (regarded as a constitutive gene) ^b^Values close to zero indicate no change respect to reference genes (*16SARNr, gyrA, dnaG*) ^c^log2ratio(M), where ratio(M) = FC ^d^log2 ratios of average FC.*

### Data Analysis

Images were captured with a GenePix 4000B (Axon) and spots quantified using GenPix software (Axon). Background correction and normalization of expression data were performed using the methods normexp and loess in LIMMA, respectively (Smyth and Speed, 2003). The expected False Discovery Rate (FDR) was controlled to be less than 5%. Genes were considered differentially expressed when nominal *p-*values were <0.05 and had a fold change (FC) equal or higher than ±2.0. FC was calculated as the average of the FC between significantly regulated probes. Hybridizations and statistical analysis were performed by the Genomics Facility at Centro Nacional de Biotecnología, CSIC, Spain.

### Microarray Data Accession Number

The microarray data provided in this study have been deposited in NCBI Gene Expression Omnibus (Edgar et al., 2002) genomics data repository and are accessible through GEO Series accession number GSE89534.

### Fatty Acid Profile of Virgin Olive Oil

Analysis of the fatty acid composition of EVOO was performed by GC after derivatization to fatty acid methyl esters (FAME). A volume of 2 mL CH3ONa 0.5 M in methanol was added to a 10–20 mg EVOO sample. The solution was vortex-shaken for 1 min and incubated 15 min at 60°C. Then, after 2 mL CH3COCl in methanol (1:10 v/v) was added, the solution was vortex-shaken for 1 min, incubated 60 min at 60°C and let stand for 10 min. Finally, 1 mL of distilled water and then 3 mL hexane were added. The solution was vortex-shaken for 1 min and centrifuged at 1500 rpm for 5 min at 4°C. The organic layer was separated and analyzed by GC.

Fatty acid methyl esters were analyzed by GC using a chromatograph (Agilent 7820A GC system) equipped with a Flame Ionization Detector (FID). Samples were introduced into a HP-23 column (60 m × 250 μm × 0.25 μm, Agilent) at 100°C and the temperature was increased at 8°C/min to 145°C (maintained for 20 min); 6°C/min to 195°C (maintained for 10 min); 3°C/min to 215°C (maintained for 5 min); 10°C/min to 230°C (maintained for 5 min). The flow rate of He, used as carrier gas, was 1 mL/min. Injector and FID temperatures were 250 and 260°C, respectively. The absolute amunt of each individual FAME was calculated by multiplying the percentage of each FAME by the amount of nonoxidized fatty acid monomers (FAM) giving results equivalent to those obtained using internal standard (C13:0).

### HPLC/ESI-MS Analysis of Olive Oil Phenolics

The phenolic fraction of EVOO samples (500 μL) was obtained by three-phase chloroform/methanol/water (1:1:1, v/v/v) extraction. The methanol/water (1:1) phase was collected, filtered through a 0.22 μm nylon membrane and directly injected into the HPLC system. Stock solutions of selected olive oil phenolics of the highest available purity (hydroxytyrosol (Seprox Biotech), *p*-coumaric acid (Sigma) and oleuropein (Extrasynthèse)) were prepared in methanol at a concentration of 20 μg/mL and stored at -20°C. Hydroxytorosol was selected as it occurs in large quantities in virgin olive oils, oleuropein as the olive precursor of hydroxytyrosol and *p*-coumaric as representative of the hydroxycinnamic acids present in olive oil. The standard solutions were prepared by serial dilution of stock solutions with water/methanol (50:50, v/v).

HPLC/ESI-MS analyses of the selected olive oil phenolics (oleuropein, hydroxytyrosol, and *p*-coumaric acid) were carried out on an Agilent 1200 chromatography system equipped with a diode array detector (DAD) and a triple quadrupole mass spectrometer (Agilent G6410B) with an electrospray interface.

Separation of phenolics was achieved on a Zorbax Eclipse Plus column (100 mm × 4.6 mm × 3.5 μm, Agilent) using gradient elution with solvent A (water/0.1% formic acid) and solvent B (acetonitrile/0.1% formic acid). The following gradient was used: 0 min 10% B; 15 min 30% B; 30 min 50% B; 32 min 10% B; 35 min 10% B. The flow rate set at 0.6 ml/min, and the injection volume was 10 μl. The analytes were quantified against authentic compounds as external standards.

## Results

### Olive Oil Characteristics

The fatty acid composition of the EVOO used in this study is shown in **Table 2**. This oil showed a fatty acid composition similar to previously reported olive oils obtained from the same olive variety (Picual) (Olivero-David et al., 2014). Oleic acid was, by far, the major fatty acid in the EVOO amounting up to 76% of the fatty acids. Overall, monounsaturated fatty acids (MUFAs) accounted for 79.5% of total fatty acid content whereas saturated fatty acids (SFAs) and polyunsaturated fatty acids (PUFAs) represented the 15.7 and 4.8% of the total FAs, respectively.

**Table 2:** Fatty acid composition and concentration of selected phenolic compounds in extra virgin olive oil (EVOO).

Fatty acid	Name	Percentage in EVOO	mg FAME/g sample
C16:0	Palmitic acid	11,27	125,41
C16:1n7	Palmitoleic acid	0,86	9,56
C17:0	Margaric acid	0,04	0,46
C18:0	Stearic acid	3,85	42,81
C18:1n7c	*Cis*-Vaccenic acid	2,42	26,92
C18:1n9c	Oleic acid	75,99	845,97
C18:2n6c	Linoleic acid	4,2	46,74
C18:3n3	α-Linolenic acid	0,61	6,81
C20:0	Arachidic acid	0,4	4,46
C20:1n9	*Cis*-11 eicosenoic acid	0,22	2,42
C22:0	Behenic acid	0,1	1,14
C24:0	Lignoceric acid	0,05	0,544
**Selected phenolic compounds (STP)**			**μg/g sample**
Hydroxytyrosol			9,31
*p*-coumaric acid			0,14
Oleuropein			n.d.

*EVOO. Extra virgin olive oil*

*n.d., Not detected.*

Regarding the phenolic compounds selected for analysis, the concentrations of hydroxytyrosol (**Table 2**) and *p*-coumaric acid (**Table 2**) found in the EVOO parallel well with concentrations previously found for these phenolics in olive oils from the same variety (Picual) (Bayram et al., 2012). As to oleuropein, it was not detected in our oil which is in line with its virtual absence or low concentration in olive oils (Perri et al., 1999) as a result of hydrolysis to yield mainly hydroxytyrosol during fruit maturation.

### Overview of the *L. plantarum* WCFS1 Transcriptomic Response to EVOO

**FIGURE 1:**
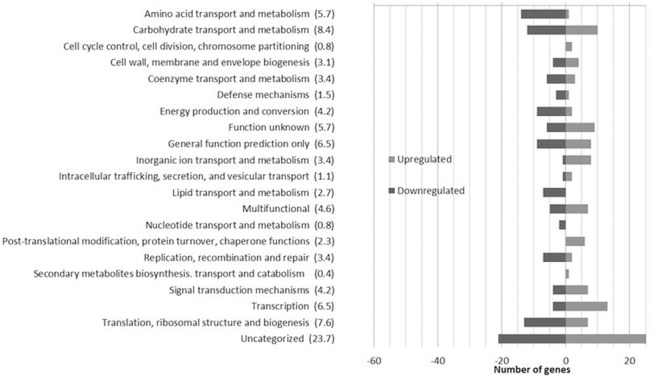
**Number of *Lactobacillus plantarum* WCFS1 genes by functional categories that were upregulated and downregulated in the presence of 50% olive oil.** The number in parentheses represents the percentage of differentially expressed genes in each category.

The impact of EVOO on the transcriptomic profile of *L. plantarum* WCFS1 was evaluated by sorting all genes whose transcript level showed changes (log_2_ratio) of at least ±2.0 (*p* < 0.05). The microarray data revealed that approximately 7% of the genes from *L. plantarum* WCFS1 were differentially expressed in presence of EVOO (**Figure 1**). Overall, 230 transcripts were affected (123 upregulated; 107 downregulated) after 10 min of exposure to EVOO as compared to transcriptomes of control cells (*t* = 0). The differentially expressed genes, functionally distributed according to their COGs categories, are shown (**Figure 1**) (Additional file 2: Supplementary Table S2).

### *L. plantarum* Downregulates the FAB Pathway during EVOO Stress

**FIGURE 2:**
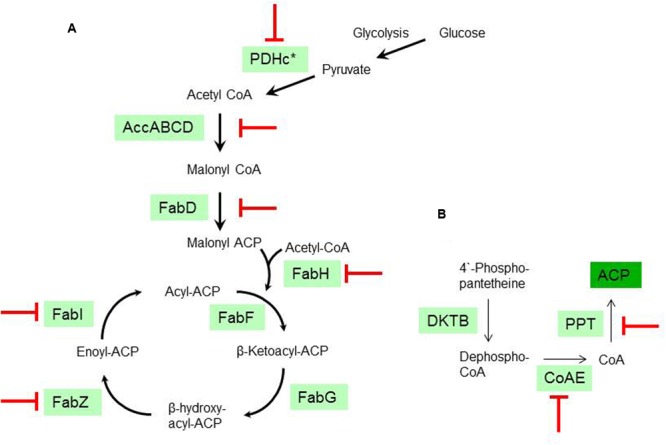
**Transcriptional inhibition of the fatty acid biosynthesis (FAB) from *L. plantarum* WCFS1 by olive oil. (A)** Schematic diagram of the type II FAB pathway according to the annotated genes in *L. plantarum* WCFS1. PDHc (PDH complex; ^∗^ see text for genes affected within this complex); PFL (pyruvate formate lyase); AccABCD (Acetyl-CoA carboxylase); FabD (Malonyl-CoA:ACP transacylase); FabH, FabF (β-Ketoacyl-ACP synthase); FabG (β-Ketoacyl-ACP reductase); FabI (enoyl-ACP reductase). **(B)** Schematic diagram of the pathway required to get the physiologically active form of the acyl carrier protein (ACP) according to the annotated genes in *L. plantarum* WCFS1. KDTB pantetheine-phosphate adenylyltransferase; CoAE (Dephospho-CoA kinase). PPT (4′-phosphopantetheinyl transferase). (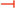), Stages transcriptionally downregulated by olive oil.

The transcriptomic response of *L. plantarum* WCFS1 to EVOO revealed the downregulation of 11 genes coding for proteins involved in various stages of the FAB pathway pathway, including the initiation (*accD1, accD2, accB3, fabD*) and elongation (*fabZ, fabI*) phases. Among the repressed genes, two coded for the E2 (*lp_2152*) and E3 (*lp_2151*) subunits of the pyruvate dehydrogenase (PDH) complex, which produces acetyl-coA to feed the FAB pathway. Further related with the PDH complex, the gene coding for lipoil protein ligase A (*lp_0477*), which is required for the lipoylation of the E2 subunit and hence essential for PDH activity, was also downregulated. In addition, genes *coaE* and *lp_1682*, which are required to get the physiologically active form of the acyl carrier protein (ACP) from 4′-phosphopantetheine, were also downregulated. For clarity, a schematic overview of the FAB pathway stages is depicted in **Figure 2**.

### Cell Wall Modification in Response to EVOO Stress

Induction of genes within the lactate racemization operon (*lp_0106, lp_0107* and *lp_0109*) and *ldhD* (D-lactate dehydrogenase), all coding for proteins involved in the synthesis of D-lactate (basic component of the *L. plantarum* peptidoglycan (PG)), was observed. In addition, genes related to transpeptidation of PG chains (*pbp2B*) and peptidoglycan hydrolases (PGHs) hydrolyzing specific PG bonds (*lytA* and *lytH*), were also upregulated. Genes involved in the decoration of the cell wall with teichoic acids (*lp_1977*, TagB primase), or in glycosylation (*lp_1763*, putative *N*-acetylgalactosamyl transferase) and mannosylation processes (*lp_1431, lp_2141*, putative dolichyl-*P*-mannose–mannosyltransferases) were downregulated by EVOO. Interestingly *lp_1643*, a gene coding for the sole mucus-binding protein among 10 new glycoproteins recently identified in *L. plantarum* (Fredriksen et al., 2013), was downregulated. Furthermore, genes coding for putative regulators of the chain length from capsular polysaccharides (*cps*) of *L. plantarum* WCFS1 were down- (*cps3E* and *cps3H*) or up-regulated (*cps4B*).

### Influence of EVOO on Osmoprotective Mechanisms

A set of seven genes involved in the transport or metabolism of compatible solutes (osmoprotectants) were differentially transcribed in the presence of EVOO. These genes included those within the trehalose operon [*lp_0262* (transcriptional repressor)], *lp_0263* (trehalose-6-phosphate hydrolase), *lp_0264* and *lp_0265* (PTS system trehalose-specific transporter subunits IIBC), the GABA transporter (*lp_1722*) and two aquaporins (*lp_0372* and *lp_3436*), which were all downregulated. This profile indicates that these compatible solutes are not required to protect *L. plantarum* from EVOO.

### Respiration Functions are Compromised by the Presence of EVOO

Genes coding for components of the electron transport chain required for the respiration metabolism of *L. plantarum* WCFS1 were down-regulated upon EVOO. Gene *ndh2* (membrane-anchored NADH-dehydrogenase), *cydB* (terminal cytochrome b), and *atpF* (ATPase B subunit) were down-regulated. In addition, other genes related to respiration metabolism such as six genes (*lp_2033, lp_2034, lp_2035, lp_2036, lp_2037, lp_1084*) involved in the synthesis of chorismate, which is the menaquinone precursor molecule, and two genes involved in the terpenoid backbone biosynthesis which are also required for menaquinone production (*lp_1732, lp_2067*), were all downregulated.

### Stress-Responsive Pathways in *L. plantarum* during EVOO-Induced Stress

Several subsets of established-responsive genes involved in countering stress were modulated by EVOO. This response included the induction of genes coding for cold-shock proteins (*lp_0031* [*cspL*], *lp_1160* [*cspP*], *lp_0997* [*cspC*]); universal stress protein (*uspA*) paralogs (*lp_1747, lp_2652*); proteases (*lp_0786* [*clpP*], *lp_1903* [*clpB*]) and a molecular chaperone (*lp_0727* [*groES*]).

Seven genes coding for proteins involved in the response to oxidative stress response in this microorganism (Serrano et al., 2007), were also induced. The encoded proteins included components of the thioredoxin-thioreductase (Trx-TrxR) system (thioredoxin (*lp_2270* [*trxA2*], *lp_2633* [*trxH*]), a glutaredoxin-like protein (*lp_0694*) [*nrdH*]) and four *spx*-encoded proteins (*lp_0836* [*spx1*], *lp_1928* [*spx2*], *lp_2228* [*spx3*], *lp_3579* [*spx5*]). Other transcriptional modifications related to countering oxidative stress included upregulation of the gene coding for the ribosomal protein 50S L31 (*lp_0512*) which has been previously reported to respond to thiol stress (Paget et al., 2001).

### Elements Typically Involved in the Stringent Response are Responsive to EVOO

Genes related to major physiological processes typically involved in the stringent response (SR) were differentially transcribed in presence of EVOO. The SR, which is triggered on exposure to nutritional or other environmental stresses in bacteria (Potrykus and Cashel, 2008), affects the expression of genes related to stress survival (such as those described above) and also of genes related to processes involved in cell proliferation.

Regarding genes involved in cell proliferative processes we observed alteration in the expression of rRNA genes which is, together with downregulation of genes related to nucleotide and FAB, the most conserved feature of the SR. From 15 rRNA genes affected, nine were downregulated (*lp_1036* [RPL2], *lp_1040* [RPS3], *lp_1041* [RPL16], *lp_1046* [RPL24], *lp_1047* [RPL5], *lp_1048* [RPS14], *lp_1052* [RPL18], *lp_1053* [RPS5], *lp_1061* [RPS11]) and six upregulated (*lp_0009* [RPS6], *lp_0512* [RPL31], *lp_0737* [RPS30A], *lp_1636* [RPS16], *lp_1973* [RPS21], *lp_2126* [RPS20]). In addition, genes coding for cell division proteins [*lp_0542* (*divIC*, septum formation initiator) and *lp_2272* (*zapA*, cell-division Z-ring component, stimulator of FtsZ polymerization)] were upregulated whereas the gene coding for the DnaB helicase (*lp_1512*), a component of the replisome, was downregulated. Noteworthy, a large accumulation of tRNA^Ser^ (*tRNA40, tRNA44*, and *tRNA50*) and at lesser extent of tRNA^Leu^ (*tRNA08*), was observed. The presence of EVOO affected the expression of five RNA modification enzymes. Two out of these genes code for RNase H activity [*lp_1853* (ribonuclease HII), *lp_2953* (ribonuclease H)], while other for exodeoxyribonuclease V (*lp_2168*). The remaining two genes encode for enzymes dedicated to tRNA modification (*lp_1579* (*miaA*)) and *lp_3688* (ribonuclease P)). Except *lp_3688* (ribonuclease P), which was upregulated, all these genes were downregulated.

The exposure to EVOO modified the expression pattern of genes involved in the *de novo* biosynthesis of purines. Thus, genes involved in the biosynthesis of ribose 5-P (which is the precursor of purines) such as *lp_2183* (ADP-ribose pyrophosphatase) and *lp_0498* (deoxyribose transporter), were downregulated. In addition, a key gene involved in folate biosynthesis (*lp_1869*, dihydrofolate reductase) and a gene requiring (tetrahydro)folate to produce formyl-methionyl tRNAf^met^ (*lp_1616*, methionyl-tRNA formyltransferase), were downregulated. Furthermore, genes coding for “Nudix” hydrolases (*lp_2826, lp_0864*), which are related to nucleotide metabolism, were up-regulated. These hydrolases are involved in the metabolism of a wide range of nucleoside 5′-diphosphate compounds (Kraszewska, 2008), including nucleotide-based secondary messengers such a (p)ppGpp or adenosine tetraphospate (Ap4A). A run in the Phyre2 modeling server ^1^ to probe the likely extent of structural similarity to other proteins showed Ap4A hydrolases as the best hits for the Nudix hydrolases encoded by *lp_2826* and *lp_0864*. Therefore, these hydrolases are potentially involved in ATP production from adenosine tetraphosphate (Ap4A).

Beside genes related to the SR, the presence of EVOO altered the expression of genes involved in the metabolism of intracellular phosphate pools, which play an important role in regulating the central metabolism of lactic acid bacteria (Levering et al., 2012). Upregulated genes within this function were *lp_0330* (fructose biphosphate aldolase), *lp_0746* (substrate binding protein of the phosphate ABC transporter), and *lp_3170* (phophoglycerate mutase). Gene *lp_1083* (transketolase, which produces glyceraldehyde-3P from ribose-5P), was downregulated.

## Discussion

**FIGURE 3:**
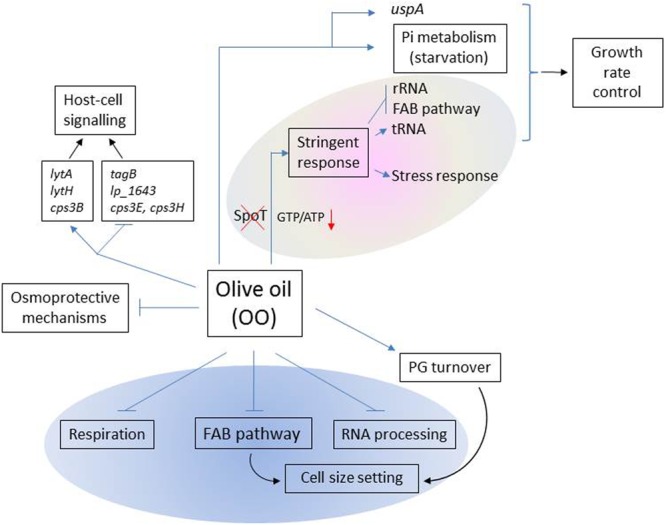
**Scheme of the main functions affected by olive oil in *L. plantarum* WCFS1.** Functions that potentially intersect with the *L. plantarum* FAB pathway are shown inside the blue oval. Within the gray oval, functions typically involved in the stringent response (SR) are represented. Blue arrows (→) represent up-regulated functons; blue perpendicular bars (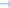), down-regulated functions.

This study provides insights on how EVOO, the main fat in the Mediterranean diet, affects the expression profile of *L. plantarum* WCFS1 at genome scale (**Figure 3**).

### Transcriptome Alterations Related to the SR

Genome-wide analyses revealed the modulation of a large number of genes typically involved in the SR. This response included the repression of genes required for cell proliferation (**Figure 3**), the accumulation of tRNA and the induction of *uspA* whose product leads to a continuous growth arrest (Kvint et al., 2003). Overall this profile is indicative of redirection of cellular resources and energy away from ribosome biosynthesis to contribute to growth rate control.

Owing to the SR, well established-responsive genes coding for proteases and molecular chaperones were also induced. A previous study has shown that cells of *Lactobacillus sakei* did not display stress responses on solid media containing olive oil (Xu et al., 2016). In the same vein transcriptomic datasets from *L. plantarum* growing in plant (carrot or pineapple) juices showed that these cells did not undergo high levels of environmental stress (Filannino et al., 2016). Although these results are in apparent contrast with the stress responses found in this study, the differences can be explained by the fact that *L. sakei* cells growing on olive oil-supplemented media or *L. plantarum* growing on plant juices were sampled after prolonged times of growth in these media (16 h or more) (Filannino et al., 2016; Xu et al., 2016). In contrast to our olive oil-misfit cells, these *Lactobacillus* cells have probably acquired some degree of adaptation, or are adapted, to thrive in the tested plant-like media at the time of sampling to perform expression studies (Filannino et al., 2016; Xu et al., 2016).

The observed transcriptional response indicated that *L. plantarum* WCFS1 challenged with EVOO underwent oxidative stress as several genes encoding for proteins involved in the thiol-specific oxidative stress response of this microorganism (Serrano et al., 2007), including up to four *spx* genes, were induced. This response might be caused by the phenolic compounds present in the EVOO, such as hydroxytytrosol or *p*-coumaric acid (**Table 2**), which may undergo auto-oxidation and provide a pro-oxidant environment. In support of this hypothesis *L. plantarum* WCFS1 also displayed a thiol-specific oxidative stress response to *p*-coumaric acid stress (Reverón et al., 2012).

The induction of the SR is widely accepted to be triggered by (p)ppGpp accumulation. The synthesis of this alarmone consumes GTP thereby resulting in a decline of GTP/ATP ratio which determines the promoter preferences of RNA polymerase. However, *L. plantarum* lacks of an SpoT homolog, which is the (p)ppGpp synthase that uniquely senses fatty acids as a signal to produce the alarmone (Hauryliuk et al., 2015). This poses the question on how *L. plantarum* can regulate the GTP/ATP ratio in response to fat stress. Regarding this issue, the transcriptomic response to EVOO revealed downregulation of genes involved in GTP biosynthetic (purine biosynthesis), and GTP-consuming pathways (such as folate biosynthesis) which may contribute to control the intracellular GTP levels, a cellular requirement for viability (Kriel et al., 2012). In the same vein, upregulation of two “Nudix” hydrolases involved in ATP production from adenosine tetraphosphate (Ap4A) may also contribute to decrease the GTP/ATP ratio (**Figure 3**).

### FAB Pathway as Potential Regulatory Hub in Biogenesis

The transcriptional response to EVOO advances knowledge on how the *L. plantarum* FAB pathway is inhibited at the transcriptional level by fatty acids (**Figure 2**). The presence of EVOO downregulated the expression of genes involved in the feeding (PDH), initiation and elongation stages of the FAB pathway (**Figure 2**). Furthermore, downregulation was extended to genes involved in the biosynthesis of cofactors (coA and lipoic acid) required to get the physiologically active form of key proteins of this pathway (ACP and PDH, respectively) (**Figure 2**). This expression profile is related to the high fatty acid content in EVOO (**Table 2**) and is consistent with the feedback inhibition of the rate of FA biosynthesis previously observed in *L. plantarum* upon exogenous addition of fatty acids to the medium (Sabaitis and Powell, 1976).

Recently, the FAB pathway has been proposed to play a central role in *Escherichia coli* (Yao et al., 2012) as well as in yeast mitochondria biogenesis (Kursu et al., 2013). FAB pathway dysfunction leads to cell size and shape alterations (Hiltunen et al., 2010; Yao et al., 2012), impairment of the respiratory function in yeast mitochondria (Hiltunen et al., 2010) and alteration of RNA processing and lipoic acid attachment to lipoic acid-dependent complexes. At present, it is unclear whether FAB pathway is important for the biogenesis of *L. plantarum*, as well. However, our transcriptional profile revealed that, besides downregulation of FAB pathway genes, similar functions of *L. plantarum* were affected by EVOO. These included the induction of genes involved in cell shape and size adjustment, down-regulation of genes coding for components of the electron transport chain required for the respiration metabolism of *L. plantarum* WCFS1 (Lechardeur et al., 2011) and the differential expression of up to five RNA processing enzymes and genes involved in the lipoic acid attachment to PDH. Interestingly, this coincidence indicates that the FAB pathway might also play a regulatory role in the biogenesis of *L. plantarum*.

### Expression Profile of Functions Related to Host-Cell Signaling

It is to note that, some of the above mentioned cell envelope functions affected at the transcriptional level by EVOO are not only involved in cell shape determination but also in the interaction with the host. For example, the PGHs encoded by the induced *lytA* and *lytH* genes generate fragments during PG cleavage that are able to modulate the host response (Rolain et al., 2012). Interestingly, other genes involved in the synthesis of surface molecules that are reported as mediators of bacteria-host interactions were also differentially regulated upon EVOO. Thus, *tagB*, a key gene in the biosynthesis of teichoic acids (which own inmunomodulatory properties) and *lp_1643*, a gene coding for a recently identified mucus binding glycoprotein (Fredriksen et al., 2013; Douillard and de Vos, 2014), were downregulated. In the same vein, modulation of genes coding for *cps* chain length regulators may have an impact in the immune response since the chain length of *cps* is an important factor for polysaccharide-induced immune response (Kalka-Moll et al., 2000). This profile is indicative that EVOO can influence the expression of metabolic traits involved in the crosstalk between this bacterium and the host (**Figure 3**).

## Conclusion

The response of *L. plantarum* WCFS1 to olive oil as studied by transcriptomics advances knowledge on the response of lactobacilli to dietary fats. The datasets revealed the involvement of elements typical of the SR in the adaptive behavior of *L. plantarum* to this fat. Expression profiling at genome scale increase our insight on how the FAB pathway is regulated in this organism and its potential intersection with functions related to cell biogenesis.

## Author Contributions

ME-T, BdR, RM, and FF conceived and designed the experiments. ME, IR, and LP performed the experiments. ME, BR, RM, and FF analyzed the data. FF drafted the manuscript. ME, BR, RM, helped to draft the manuscript. All authors read and approved the final manuscript.

## Conflict of Interest Statement

The authors declare that the research was conducted in the absence of any commercial or financial relationships that could be construed as a potential conflict of interest.

## Footnotes

1 http://www.sbg.bio.ic.ac.uk/~phyre/
