# Supplementary material for: Transcriptional Reprogramming at Genome-Scale of Lactobacillus plantarum WCFS1 in Response to Olive Oil Challenge
Source: Front Microbiol. 2017 Feb 17;8:244. doi: 10.3389/fmicb.2017.00244 (PMC5313477; doi:10.3389/fmicb.2017.00244)
Supplement: Supplementary file 1 [file Table_1.DOC]

**Additional file 1: Table S1.** Oligonucleotides used for qRT-PCR in this study

| **Locus Tag^a^** | **Description** | **Forward primer sequence** | **Reverse primer sequence** | **References** |
| --- | --- | --- | --- | --- |
| *lp_0009* | 30S ribosomal protein S6 | TGCGCAAGTAATTGATTCAAAAG | AAGCCACCGATTTCGTATGC | This study |
| *lp_0265* | PTS system trehalose-specific transporter subunit IIBC | AGCGCACGATGCGGTTA | GGCCTTTGCAACATCAATGA | This study |
| *lp_0997* | Cold shock protein CspC | TCGTACATTTCTCAGCTATCCAAGA | GTTTACAGCTTGGCCTTCATCA | This study |
| *lp_1449* | Cell surface protein, CscB family | TGACTGGTGCGGTGATTCA | CGGGCTCGTTTAGCGAATTA | This study |
| *lp_1880* | Hypothetical protein | GCATTCCTATGCTATCTTTGATGAAG | GATGATCATTTTTGAGTGGTCCAA | This study |
| *lp_2035* | 3-phosphoshikimate 1-carboxyvinyltransferase | GGATGGGCGAACCAGTTG | CCGGATGCAGTTGTGCAA | This study |
| *lp_2368* | H(+)-transporting two-sector ATPase, B subunit | GCTTGGAAGCCCATCACAAA | CAATGTCGTTCGCAATCTTGTC | This study |
| *lp_2755* | Membrane protein | GGGCCAGCCAAAAACAAA | GCCAAGGCACCTAATGTATTCAT | This study |
| **Housekeeping and other internal control genes:** | |  |  |  |
| *lp_2057* | D-lactate dehydrogenase | AACCGCGACAATGTTTTGATT | TTGTGAACGGCAGTTTCAGTGT | Reverón *et al*., 2013 |
| *lp_1963^c^* | DNA primase DnaG | TCCGGAAGCAGTCGTCAAG | TCGCCGGCAAGTCAATGT | Reverón *et al*., 2013 |
| *lp_0007* | DNA gyrase, A subunit | CCCGACAGCAACGTCTTCA | GGCAGCTGGCGTTTGTTT | Reverón *et al*., 2013 |
| *lp_1962* | RNA polymerase sigma factor RpoD | CGGATCCGCCAAATCG | CGTGATGGGTGGCGTAACTT | Reverón *et al*., 2013 |
| *lp_2301* | recombinase A | CGGCGGGCAGAACAGAT | TTTCCAAGCCACTCTTTTTTCG | Reverón *et al*., 2013 |
| *lp_0789* | Glyceraldehyde 3-phosphate dehydrogenase | CTGGTGCTGCTAAGGCTCTTG | TGTGCATGGCCTTGTAATTTACC | Reverón *et al*., 2015 |
| *lp_rRNA01* | 16S ribosomal rRNA | GGGTAATCGGCCACATTGG | CTGCTGCCTCCCGTAGGA | Reverón *et al*., 2013 |

**^a^** Designated gene number for the annotated *L. plantarum* WCFS1 genome.

**^b^** Internal control gene used to calculate the relative expression.
