# Supplementary material for: Transcriptional Reprogramming at Genome-Scale of Lactobacillus plantarum WCFS1 in Response to Olive Oil Challenge
Source: Front Microbiol. 2017 Feb 17;8:244. doi: 10.3389/fmicb.2017.00244 (PMC5313477; doi:10.3389/fmicb.2017.00244)
Supplement: Supplementary file 2 [file Table_2.DOCX]

**Additional file 2. Table S2.** *Lactobacillus plantarum* WCFS1 genes with differential expression in presence of olive oil 50 %

| Locus Tag | Locus | Description | COG main functional category | Fold  Change ^a,b^ | Subcellular Localization Prediction ^c^ | Pathway Prediction |
| --- | --- | --- | --- | --- | --- | --- |
| *lp_0009* | *rpsF* | 30S ribosomal protein S6 | J: Translation, ribosomal structure and biogenesis | 2,99 | Intracellular |  |
| *lp_0031* | *cspL* | cold shock protein CspL | K: Transcription | 2,05 | Intracellular |  |
| *lp_0068* |  | Membrane protein | S: Function unknown | -1,98 | Multi-transmembrane | Sec-(SPI) |
| *lp_0074* |  | DeoR family transcriptional regulator | K: Transcription | 2,04 | Intracellular |  |
| *lp_0091* |  | pyridoxamine 5\'-phosphate oxidase family protein, FMN-binding |  | 2,55 | Intracellular |  |
| *lp_0106* | *larC1* | lactate racemization operon protein LarC,N-terminal domain |  | 2,78 |  |  |
| *lp_0107* | *larC2* | lactate racemization operon protein LarC,C-terminal domain |  | 2,5 |  |  |
| *lp_0109* | *larE* | lactate racemization operon protein LarE | R: General function prediction only | 2,53 | Intracellular |  |
| *lp_0126* |  | stress-responsive transcription regulator | K: Transcription | 3,08 | N-terminally anchored (No CS) | Sec-(SPI) |
|  |  |  | T: Signal transduction mechanisms |  |  |  |
| *lp_0130* |  | two-component system sensor protein |  | 2,16 | Multi-transmembrane | Sec-(SPI) |
| *lp_0141* |  | extracellular protein |  | 2,55 | Secretory (released) (with CS) | Sec-(SPI) |
| *lp_0183* |  | membrane protein |  | 2,37 | Multi-transmembrane | Sec-(SPI) |
| *lp_0237* |  | membrane protein |  | 2,42 | Multi-transmembrane | Sec-(SPI) |
| *lp_0245* |  | GntR family transcriptional regulator | K: Transcription | 2,1 | Intracellular |  |
| *lp_0247* | *pts3C* | PTS system, cellobiose-specific EIIC component | G: Carbohydrate transport and metabolism | 2,37 | Multi-transmembrane | Sec-(SPI) |
| *lp_0262* | *treR* | trehalose operon transcriptional repressor,GntR family | K: Transcription | -2,22 | Intracellular |  |
| *lp_0263* | *treA* | trehalose-6-phosphate hydrolase | G: Carbohydrate transport and metabolism | -2,08 | Intracellular |  |
| *lp_0264* | *pts4ABC* | PTS system trehalose-specific transporter subunit IIBC | G: Carbohydrate transport and metabolism | -2,22 | Multi-transmembrane | Sec-(SPI) |
| *lp_0265* | *pts5ABC* | PTS system trehalose-specific transporter subunit IIBC | G: Carbohydrate transport and metabolism | -4,47 | Intracellular/TMH start after 60 | Possibly Sec- |
| *lp_0279* |  | hypothetical protein lp_0279 |  | 2,31 | Intracellular |  |
| *lp_0301* |  | membrane-bound protease, CAAX family |  | 2,22 | Multi-transmembrane | Sec-(SPI) |
| *lp_0312* |  | MarR family transcriptional regulator | K: Transcription | 2,27 | Intracellular |  |
| *lp_0315* | *potD* | spermidine/putrescine ABC transporter,substrate binding protein | E: Amino acid transport and metabolism | -2,1 | N-terminally anchored (No CS) | Sec-(SPI) |
| *lp_0319* |  | spermidine/putrescine transport operon transcriptional regulator | K: Transcription | 2,21 | Intracellular |  |
| *lp_0330* | *fba* | fructose-bisphosphate aldolase | G: Carbohydrate transport and metabolism | 3,19 | Intracellular |  |
| *lp_0346* |  | membrane protein |  | 2,33 | Multi-transmembrane | Sec-(SPI) |
| *lp_0357* |  | membrane protein | S: Function unknown | 2,33 | Multi-transmembrane | Sec-(SPI) |
| *lp_0362* | *accB3* | acetyl-CoA carboxylase, biotin carboxyl carrier protein | I: Lipid transport and metabolism | -2,01 | Intracellular |  |
| *lp_0372* | *glpF3* | glycerol uptake facilitator protein | G: Carbohydrate transport and metabolism | -2,53 | Multi-transmembrane | Sec-(SPI) |
| *lp_0391* |  | membrane protein |  | 2,24 | Multi-transmembrane | Sec-(SPI) |
| *lp_0402* |  | hypothetical protein lp_0402 |  | 2,25 | Intracellular |  |
| *lp_0406* | *plnJ* | bacteriocin precursor peptide PlnJ |  | 2,76 | Secreted via minor pathways (bacteriocin) (no CS) | Non-classical |
| *lp_0422* | *plnE* | bacteriocin precursor peptide PlnE |  | 2,13 | Secreted via minor pathways (bacteriocin) (no CS) | Non-classical |
| *lp_0438* |  | hypothetical protein lp_0438 |  | -2,37 | Intracellular |  |
| *lp_0477* | *mvaA* | lipoate-protein ligase A | H: Coenzyme transport and metabolism | -2,11 | Intracellular |  |
| *lp_0498* | *deoP* | deoxyribose transporter | G: Carbohydrate transport and metabolism | -2 | Multi-transmembrane | Sec-(SPI) |
| *lp_0509* |  | hypothetical protein lp_0509 |  | 2,35 | Intracellular |  |
| *lp_0512* | *rpmE* | 50S ribosomal protein L31 | J: Translation, ribosomal structure and biogenesis | 2,87 | Intracellular |  |
| *lp_0535* |  | hypothetical protein lp_0535 | T: Signal transduction mechanisms | -2,06 | Intracellular |  |
| *lp_0540* |  | polysaccharide transporter | R: General function prediction only | -2,11 | Multi-transmembrane | Sec-(SPI) |
| *lp_0542* | *divIC* | septum formation initiator | D: Cell cycle control, cell division, chromosome partitioning | 2,14 | N-terminally anchored (No CS) | Sec-(SPI) |
| *lp_0551* | *dtpT* | di-/tripeptide transport protein | E: Amino acid transport and metabolism | -2,26 | Multi-transmembrane | Sec-(SPI) |
| *lp_0565* | *nadC1* | nicotinate phosphoribosyltransferase | H: Coenzyme transport and metabolism | 2,02 | Intracellular |  |
| *lp_0583* |  | hypothetical protein lp_0583 | S: Function unknown | 2,06 | Intracellular |  |
| *lp_0591* | *accD1* | acetyl-CoA carboxylase, carboxyl transferase subunit beta | I: Lipid transport and metabolism | -2,17 | Intracellular |  |
| *lp_0593* |  | transcriptional attenuator, cell envelope-related, LytR family | K: Transcription | 2,05 | N-terminally anchored (No CS) | Sec-(SPI) |
| *lp_0616* | *secE* | Preprotein translocase subunit SecE | U: Intracellular trafficking, secretion, and vesicular transport | 2,42 | N-terminally anchored (No CS) | Sec-(SPI) |
| *lp_0686* |  | prophage P1 protein 63 |  | 2,26 |  |  |
| *lp_0694* | *nrdH* | glutaredoxin-like protein nrdH | O: Posttranslational modification, protein turnover, chaperones | 2,29 | Intracellular |  |
| *lp_0727* | *groES* | GroES co-chaperonin | O: Posttranslational modification, protein turnover, chaperones | 2,22 | Intracellular |  |
| *lp_0737* |  | ribosomal protein S30EA | J: Translation, ribosomal structure and biogenesis | 2,74 | Intracellular |  |
| *lp_0746* | *pstE* | phosphate ABC transporter substrate-binding protein | P: Inorganic ion transport and metabolism | 3,21 | N-terminally anchored (No CS) | Sec-(SPI) |
| *lp_0769* | *aad* | D-alanyl-D-alanine dipeptidase | M: Cell wall/membrane/envelope biogenesis | -1,96 | Intracellular |  |
| *lp_0786* | *clpP* | endopeptidase Clp, proteolytic subunit | O: Posttranslational modification, protein turnover, chaperones | 2,12 | Intracellular |  |
|  |  |  | U: Intracellular trafficking, secretion, and vesicular transport |  |  |  |
| *lp_0807* | *pta* | phosphate acetyltransferase | C: Energy production and conversion | -1,95 | Intracellular |  |
| *lp_0827* |  | hypothetical protein lp_0827 |  | 2,48 | Intracellular |  |
| *lp_0836* | *spx1* | RNA polymerase (RNAP)-binding regulatory protein, arsenate reductase (ArsC) family, Spx subfamily | P: Inorganic ion transport and metabolism | 3,11 | Intracellular |  |
| *lp_0837* |  | hypothetical protein lp_0837 |  | 2,52 | Intracellular |  |
| *lp_0864* |  | phosphohydrolase |  | 2,41 | Intracellular |  |
| *lp_0865* |  | hypothetical protein lp_0865 |  | 1,95 | Intracellular |  |
| *lp_0868* |  | metal uptake regulator | P: Inorganic ion transport and metabolism | 2,15 | Intracellular |  |
| *lp_0875* |  | hypothetical protein lp_0875 |  | 2,18 | Intracellular |  |
| *lp_0910* |  | DNA helicase | R: General function prediction only | 2,09 | Intracellular |  |
| *lp_0919* |  | membrane-bound protease, CAAX family |  | -2,36 | Multi-transmembrane | Sec-(SPI) |
| *lp_0992* |  | MerR family transcriptional regulator | K: Transcription | 2,04 | Intracellular |  |
| *lp_0995* |  | hypothetical protein lp_0995 |  | 2,41 | Intracellular |  |
| *lp_0997* | *cspC* | cold shock protein CspC | K: Transcription | 3,61 | Intracellular |  |
| *lp_1036* | *rplB* | 50S ribosomal protein L2 | J: Translation, ribosomal structure and biogenesis | -2,39 | Intracellular |  |
| *lp_1040* | *rpsC* | 30S ribosomal protein S3 | J: Translation, ribosomal structure and biogenesis | -2,33 | Intracellular |  |
| *lp_1041* | *rplP* | 50S ribosomal protein L16 | J: Translation, ribosomal structure and biogenesis | -1,96 | Intracellular |  |
| *lp_1046* | *rplX* | 50S ribosomal protein L24 | J: Translation, ribosomal structure and biogenesis | -1,94 | Intracellular |  |
| *lp_1047* | *rplE* | 50S ribosomal protein L5 | J: Translation, ribosomal structure and biogenesis | -2,07 | Intracellular |  |
| *lp_1048* | *rpsN* | 30S ribosomal protein S14 | J: Translation, ribosomal structure and biogenesis | -2,61 | Intracellular |  |
| *lp_1052* | *rplR* | 50S ribosomal protein L18 | J: Translation, ribosomal structure and biogenesis | -2,18 | Intracellular |  |
| *lp_1053* | *rpsE* | 30S ribosomal protein S5 | J: Translation, ribosomal structure and biogenesis | -2,02 | Intracellular |  |
| *lp_1058* | *adk* | adenylate kinase | F: Nucleotide transport and metabolism | -2,41 | Intracellular |  |
| *lp_1061* | *rpsK* | 30S ribosomal protein S11 | J: Translation, ribosomal structure and biogenesis | -2,22 | Intracellular |  |
| *lp_1069* | *ndh2* | NADH dehydrogenase, membrane-anchored | C: Energy production and conversion | -3,2 | Multi-transmembrane | Sec-(SPI) |
| *lp_1083* | *tkt2* | transketolase | G: Carbohydrate transport and metabolism | -2,55 | Intracellular |  |
| *lp_1084* | *aroD1* | shikimate 5-dehydrogenase | E: Amino acid transport and metabolism | -2,31 | Intracellular |  |
| *lp_1086* | *aroB* | 3-dehydroquinate synthase | E: Amino acid transport and metabolism | -2,1 | Intracellular |  |
| *lp_1095* | *mtsC* | manganese ABC transporter, ATP-binding protein | P: Inorganic ion transport and metabolism | 2,35 | Intracellular |  |
| *lp_1126* | *cydB* | cytochrome D ubiquinol oxidase subunit II | C: Energy production and conversion | -1,99 | Multi-transmembrane | Sec-(SPI) |
| *lp_1160* | *cspP* | cold shock protein CspP | K: Transcription | 3,29 | Intracellular |  |
| *lp_1163* |  | nucleotide-binding protein, universal stress protein UspA family | T: Signal transduction mechanisms | -2,52 | Intracellular |  |
| *lp_1164* | *pts14C* | PTS system, cellobiose-specific EIIC component | G: Carbohydrate transport and metabolism | -2,01 | Multi-transmembrane | Sec-(SPI) |
| *lp_1168* |  | hypothetical protein lp_1168 |  | -2,54 | Intracellular |  |
| *lp_1200* | *cps2D* | UDP N-acetyl glucosamine 4-epimerase, NAD dependent | M: Cell wall/membrane/envelope biogenesis | 2,06 | Intracellular |  |
|  |  |  | G: Carbohydrate transport and metabolism |  |  |  |
| *lp_1221* | *cps3E* | polysaccharide biosynthesis protein |  | -2,98 | Multi-transmembrane | Sec-(SPI) |
| *lp_1225* | *cps3H* | polysaccharide biosynthesis protein |  | -2,21 | Multi-transmembrane | Sec-(SPI) |
| *lp_1249* | *gntP* | gluconate transport protein | G: Carbohydrate transport and metabolism | -3,18 | Multi-transmembrane | Sec-(SPI) |
|  |  |  | E: Amino acid transport and metabolism |  |  |  |
| *lp_1262* | *oppB* | oligopeptide ABC transporter permease | E: Amino acid transport and metabolism | -2,03 | Multi-transmembrane | Sec-(SPI) |
|  |  |  | P: Inorganic ion transport and metabolism |  |  |  |
| *lp_1277* |  | membrane protein | S: Function unknown | -2,16 | Multi-transmembrane | Sec-(SPI) |
| *lp_1281* |  | hypothetical protein lp_1281 | S: Function unknown | 3,63 | Intracellular |  |
| *lp_1321* | *pepV* | Xaa-His dipeptidase | E: Amino acid transport and metabolism | 2,06 | Intracellular |  |
| *lp_1335* |  | ABC transporter ATP-binding protein | V: Defense mechanisms | -2,02 | Intracellular |  |
| *lp_1336* |  | bifunctional protein: ABC transporter ATP-binding protein; transcription regulator, LytR family | V: Defense mechanisms | -2,03 | Intracellular |  |
| *lp_1363* |  | hypothetical protein lp_1363 |  | 2,14 | Intracellular |  |
| *lp_1431* |  | membrane protein |  | -2,57 | Multi-transmembrane | Sec-(SPI) |
| *lp_1449* |  | cell surface protein, CscB family |  | -2,59 | Secretory(released) (with CS) | Sec-(SPI) |
| *lp_1456* |  | ABC transporter permease | U: Intracellular trafficking, secretion, and vesicular transport | -2,41 | Multi-transmembrane | Sec-(SPI) |
| *lp_1467* | *feoA* | ferrous iron transport protein A | P: Inorganic ion transport and metabolism | 2,17 | Intracellular |  |
| *lp_1509* | *fpg* | formamidopyrimidine-DNA glycosylase | L: Replication, recombination and repair | -2,02 | Intracellular |  |
| *lp_1510* | *coaE* | dephospho-CoA kinase | H: Coenzyme transport and metabolism | -2,51 | N-terminally anchored (No CS) | Sec-(SPI) |
| *lp_1512* | *dnaB* | replication initiation and membrane attachment protein DnaB | L: Replication, recombination and repair | -2,77 | Intracellular |  |
| *lp_1530* | *nadD* | nicotinate-nucleotide adenylyltransferase | H: Coenzyme transport and metabolism | -2,36 | Intracellular |  |
| *lp_1532* |  | hypothetical protein lp_1532 | S: Function unknown | -1,94 | Intracellular |  |
| *lp_1533* |  | methyltransferase | H: Coenzyme transport and metabolism | -2,15 | Intracellular |  |
| *lp_1534* |  | hypothetical protein lp_1534 | R: General function prediction only | -2,13 | Intracellular |  |
| *lp_1564* |  | membrane protein |  | -2,21 | Multi-transmembrane | Sec-(SPI) |
| *lp_1565* |  | LytTr family transcriptional regulator | K: Transcription | -2,41 | Intracellular |  |
|  |  |  | T: Signal transduction mechanisms |  |  |  |
| *lp_1566* |  | hypothetical protein lp_1566 | S: Function unknown | 2,07 | N-terminally anchored (No CS) | Sec-(SPI) |
| *lp_1568* | *pbp2x* | transpeptidase (penicillin binding protein 2B) | M: Cell wall/membrane/envelope biogenesis | 2,98 | N-terminally anchored (No CS) | Sec-(SPI) |
| *lp_1577* |  | hypothetical protein lp_1577 |  | 3,17 | N-terminally anchored (No CS) | Sec-(SPI) |
| *lp_1579* | *miaA* | tRNA isopentenylpyrophosphate transferase | J: Translation, ribosomal structure and biogenesis | -2,22 | N-terminally anchored (No CS) | Sec-(SPI) |
| *lp_1615* | *priA* | helicase, primosomal protein N\' | L: Replication, recombination and repair | -2,13 | Intracellular |  |
| *lp_1616* | *fmt* | methionyl-tRNA formyltransferase | J: Translation, ribosomal structure and biogenesis | -1,99 | N-terminally anchored (No CS) | Sec-(SPI) |
| *lp_1636* | *rpsP* | 30S ribosomal protein S16 | J: Translation, ribosomal structure and biogenesis | 2,15 | Intracellular |  |
| *lp_1643* |  | mucus-binding protein precursor, LPXTG-motif cell wall anchor |  | -2,15 | LPxTG Cell-wall anchored | Sec-(SPI) |
| *lp_1673* | *fabD* | [acyl-carrier protein] S-malonyltransferase [Lactobacillus plantarum WCFS1] | I: Lipid transport and metabolism | -2,46 | Intracellular |  |
| *lp_1677* | *fabZ2* | (3R)-hydroxymyristoyl-ACP dehydratase | I: Lipid transport and metabolism | -2,41 | Intracellular |  |
| *lp_1679* | *accD2* | acetyl-CoA carboxylase, carboxyl transferase subunit beta | I: Lipid transport and metabolism | -2,44 | Intracellular |  |
| *lp_1681* | *fabI* | enoyl | I: Lipid transport and metabolism | -2,25 | Intracellular |  |
| *lp_1682* |  | phosphopantetheinyltransferase | H: Coenzyme transport and metabolism | -2,23 | Intracellular |  |
| *lp_1687* | *rsgA* | ribosome small subunit-dependent GTPase A | R: General function prediction only | -2,68 | Intracellular |  |
| *lp_1699* |  | hypothetical protein lp_1699 |  | 3,07 | Intracellular |  |
| *lp_1700* |  | membrane protein | T: Signal transduction mechanisms | -2,49 | Multi-transmembrane | Sec-(SPI) |
| *lp_1711* | *pepD4* | dipeptidase | E: Amino acid transport and metabolism | -2,35 | N-terminally anchored (No CS) | Sec-(SPI) |
| *lp_1712* | *xylH* | 4-oxalocrotonate tautomerase | R: General function prediction only | 2,71 | Intracellular |  |
| *lp_1722* |  | 4-aminobutanoate transport protein | E: Amino acid transport and metabolism | -2,31 | Multi-transmembrane | Sec-(SPI) |
| *lp_1726* |  | hypothetical protein lp_1726 |  | -2,03 | Intracellular /TMH start AFTER 60 | Possibly Sec- |
| *lp_1732* | *idi1* | isopentenyl diphosphate delta-isomerase | C: Energy production and conversion | -2,63 | Intracellular |  |
| *lp_1747* |  | nucleotide-binding protein, universal stress protein UspA family | T: Signal transduction mechanisms | 2,17 | Intracellular |  |
| *lp_1763* |  | glycosyltransferase | M: Cell wall/membrane/envelope biogenesis | -2,25 | Intracellular |  |
| *lp_1774* |  | alpha/beta hydrolase | R: General function prediction only | -2,04 | Intracellular |  |
| *lp_1803* |  | transport protein, major facilitator subfamily (MFS) | G: Carbohydrate transport and metabolism | -2,29 | Multi-transmembrane | Sec-(SPI) |
| *lp_1812* |  | lipoprotein |  | 0,12 | Lipid anchored | Sec-(SPII) |
| *lp_1814* |  | L-lactate transport protein | C: Energy production and conversion | -2,21 | Multi-transmembrane | Sec-(SPI) |
| *lp_1815* |  | membrane protein |  | -2,25 | Multi-transmembrane | Sec-(SPI) |
| *lp_1853* | *rnhB* | ribonuclease HII | L: Replication, recombination and repair | -2,41 | Intracellular |  |
| *lp_1869* | *dfrA* | dihydrofolate reductase | H: Coenzyme transport and metabolism | -2,15 | Intracellular |  |
| *lp_1872* |  | hypothetical protein lp_1872 |  | 2,18 | Intracellular |  |
| *lp_1880* |  | hypothetical protein lp_1880 |  | 4,95 | Intracellular |  |
| *lp_1886* |  | hypothetical protein lp_1886 | S: Function unknown | -2,24 | Intracellular |  |
| *lp_1903* | *clpB* | ATP-dependent Clp protease, ATP-binding subunit ClpB | O: Posttranslational modification, protein turnover, chaperones | 2,18 | Intracellular |  |
| *lp_1908* |  | membrane protein |  | -2,62 | Multi-transmembrane | Sec-(SPI) |
| *lp_1928* | *spx2* | RNA polymerase (RNAP)-binding regulatory protein, arsenate reductase (ArsC) family, Spx subfamily | P: Inorganic ion transport and metabolism | 2,2 | Intracellular |  |
| *lp_1939* |  | oxidoreductase, medium chain dehydrogenases/reductase (MDR)/zinc-dependent alcohol dehydrogenase-lik... | C: Energy production and conversion | -2,36 | Intracellular |  |
|  |  |  | R: General function prediction only |  |  |  |
| *lp_1953* |  | hypothetical protein lp_1953 |  | 2,36 | Intracellular |  |
| *lp_1956* |  | ABC transporter permease |  | -2,22 | Multi-transmembrane | Sec-(SPI) |
| *lp_1958* |  | acetoin ABC transporter, ATP-binding protein | V: Defense mechanisms | 1,97 | Intracellular |  |
| *lp_1966* | *recO* | DNA repair protein RecO | L: Replication, recombination and repair | -2,01 | Intracellular |  |
| *lp_1972* |  | hypothetical protein lp_1972 | S: Function unknown | 2,8 | Intracellular |  |
| *lp_1973* | *rpsU* | 30S ribosomal protein S21 | J: Translation, ribosomal structure and biogenesis | 3,55 | Intracellular |  |
| *lp_1974* |  | transcription regulator of gluconeogenic genes | S: Function unknown | -2,09 | Intracellular |  |
| *lp_1977* | *tagB3* | glycosyl/glycerophosphate transferase, teichoic acid biosynthesis protein B precursor | M: Cell wall/membrane/envelope biogenesis | -2,43 | Intracellular |  |
| *lp_1982* | *lytH* | N-acetylmuramoyl-L-alanine amidase | M: Cell wall/membrane/envelope biogenesis | 2,08 | N-terminally anchored (No CS) | Sec-(SPI) |
| *lp_1992* |  | hypothetical protein lp_1992 |  | 2,23 | Intracellular |  |
| *lp_2004* |  | membrane protein |  | -2,2 | Multi-transmembrane | Sec-(SPI) |
| *lp_2033* | *aroI* | shikimate kinase | E: Amino acid transport and metabolism | -2,51 | Intracellular |  |
| *lp_2034* | *tyrA* | prephenate dehydrogenase | E: Amino acid transport and metabolism | -2,7 | Intracellular |  |
| *lp_2035* | *aroE* | 3-phosphoshikimate 1-carboxyvinyltransferase | E: Amino acid transport and metabolism | -2,79 | Intracellular |  |
| *lp_2036* |  | aromatic amino acid biosynthesis enzyme |  | -2,22 | Intracellular |  |
| *lp_2037* | *aroF* | chorismate synthase | E: Amino acid transport and metabolism | -2,58 | Intracellular |  |
| *lp_2057* | *ldhD* | D-lactate dehydrogenase | C: Energy production and conversion | 2,79 | Intracellular |  |
|  |  |  | H: Coenzyme transport and metabolism |  |  |  |
|  |  |  | R: General function prediction only |  |  |  |
| *lp_2063* | *lexA* | transcription repressor and protease LexA of the SOS regulon | K: Transcription | 2,67 | Intracellular |  |
|  |  |  | T: Signal transduction mechanisms |  |  |  |
| *lp_2067* | *mvaS* | hydroxymethylglutaryl-CoA synthase | I: Lipid transport and metabolism | -2,5 | Intracellular |  |
| *lp_2107* | *cps4B* | polysaccharide biosynthesis protein | D: Cell cycle control, cell division, chromosome partitioning | 3,14 | Intracellular |  |
| *lp_2112* |  | hypothetical protein lp_2112 |  | 3,05 | Intracellular |  |
| *lp_2119* | *tuf* | translation elongation factor Tu | J: Translation, ribosomal structure and biogenesis | 2,25 | Intracellular |  |
| *lp_2126* | *rpsT* | 30S ribosomal protein S20 | J: Translation, ribosomal structure and biogenesis | 3,51 | Intracellular |  |
| *lp_2141* |  | membrane protein |  | -2,49 | Multi-transmembrane | Sec-(SPI) |
| *lp_2151* | *pdhD* | pyruvate dehydrogenase complex, E3 component; dihydrolipoamide dehydrogenase | C: Energy production and conversion | -3,18 | Intracellular |  |
| *lp_2152* | *pdhC* | pyruvate dehydrogenase complex, E2 component; dihydrolipoamide S-acetyltransferase | C: Energy production and conversion | -2,14 | Intracellular |  |
| *lp_2168* | *recD* | exodeoxyribonuclease V, alpha chain | L: Replication, recombination and repair | -2,04 | Intracellular |  |
| *lp_2183* |  | ADP-ribose pyrophosphatase | F: Nucleotide transport and metabolism | -2,06 | Intracellular |  |
| *lp_2221* | *rluD2* | pseudouridylate synthase | J: Translation, ribosomal structure and biogenesis | -2,22 | Intracellular |  |
| *lp_2226* |  | competence protein/transcription factor,CoiA-like family | R: General function prediction only | -2,27 | Intracellular |  |
| *lp_2228* | *spx3* | RNA polymerase (RNAP)-binding regulatory protein, arsenate reductase (ArsC) family, Spx subfamily | P: Inorganic ion transport and metabolism | 2,39 | Intracellular |  |
| *lp_2254* |  | hypothetical protein lp_2254 | S: Function unknown | -2,06 | Intracellular |  |
| *lp_2270* | *trxA2* | thioredoxin | O: Posttranslational modification, protein turnover, chaperones | 2,22 | Intracellular |  |
| *lp_2272* | *zapA* | cell-division Z-ring component, stimulator of FtsZ polymerization |  | 2,33 | Intracellular |  |
| *lp_2275* |  | hypothetical protein lp_2275 | S: Function unknown | 2,13 | Intracellular |  |
| *lp_2339* |  | membrane protein |  | 2,34 | Multi-transmembrane | Sec-(SPI) |
| *lp_2347* |  | hypothetical protein lp_2347 |  | -2,21 | Intracellular |  |
| *lp_2368* | *atpF* | H(+)-transporting two-sector ATPase, B subunit | C: Energy production and conversion | -2,72 | N-terminally anchored (No CS) | Sec-(SPI) |
| *lp_2377* |  | S-adenosyl-L-methionine-dependent N(5)-glutamine methyltransferase, release factor-specific | J: Translation, ribosomal structure and biogenesis | -1,98 | Intracellular |  |
| *lp_2410* |  | prophage P2a protein 47 |  | -2,04 | Intracellular |  |
| *lp_2443* |  | hypothetical protein lp_2443A/hypothetical protein lp_2443B | S: Function unknown | 3,26 | Intracellular |  |
| *lp_2460* |  | prophage P2b protein 21 |  | 2,85 | Multi-transmembrane | Sec-(SPI) |
| *lp_2467* |  | prophage P2b protein 14, terminase small subunit | L: Replication, recombination and repair | 2,17 | Intracellular |  |
| *lp_2593* | *rnh* | ribonuclease H | L: Replication, recombination and repair | -2,04 | Intracellular |  |
| *lp_2633* | *trxH* | thioredoxin H-type | O: Posttranslational modification, protein turnover, chaperones | 2,06 | Intracellular |  |
| *lp_2648* | *pts19D* | PTS system, N-acetylglucosamine-specific EIID component | G: Carbohydrate transport and metabolism | -2,63 | Multi-transmembrane | Sec-(SPI) |
| *lp_2649* | *pts19C* | PTS system, N-acetylglucosamine-specific EIIC component | G: Carbohydrate transport and metabolism | -2,25 | Multi-transmembrane | Sec-(SPI) |
| *lp_2652* |  | nucleotide-binding protein, universal stress protein UspA family | T: Signal transduction mechanisms | 1,95 | Intracellular |  |
| *lp_2658* |  | glycosyltransferase, family 1 (GT1) | M: Cell wall/membrane/envelope biogenesis | -2,39 | Intracellular |  |
| *lp_2671* |  | transport protein with CBS domains | R: General function prediction only | 2,01 | Multi-transmembrane | Sec-(SPI) |
| *lp_2755* |  | membrane protein |  | 2,67 | Multi-transmembrane | Sec-(SPI) |
| *lp_2758* | *thrC* | threonine synthase | E: Amino acid transport and metabolism | -2,11 | Intracellular |  |
| *lp_2781* | *pts20B* | PTS system, cellobiose-specific EIIB component | G: Carbohydrate transport and metabolism | 1,98 | N-terminally anchored (No CS) | Sec-(SPI) |
| *lp_2823* |  | ABC transporter ATP-binding protein | V: Defense mechanisms | -2,96 | Intracellular |  |
| *lp_2826* |  | dihydrofolate reductase family protein | H: Coenzyme transport and metabolism | 2,33 | Intracellular |  |
| *lp_2829* | *mleP3* | malate transport protein | R: General function prediction only | -1,98 | Multi-transmembrane | Sec-(SPI) |
| *lp_2861* | *thgA3* | galactoside O-acetyltransferase | R: General function prediction only | 2,42 | Intracellular |  |
| *lp_2868* | *pgm8* | phosphoglycerate mutase family protein | G: Carbohydrate transport and metabolism | -1,98 | Intracellular |  |
| *lp_2922* | *npp* | nucleotide pyrophosphatase | R: General function prediction only | 2,34 | Intracellular |  |
| *lp_2968* |  | nitroreductase | C: Energy production and conversion | 2,17 | Intracellular |  |
| *lp_3013* |  | MerR family transcriptional regulator | K: Transcription | 2,06 | Intracellular |  |
| *lp_3025* |  | extracellular protein, membrane-anchored |  | 2,18 | N-terminally anchored (No CS) | Sec-(SPI) |
| *lp_3116* |  | cell surface protein, CscB family |  | -2,07 | Secretory(released) (with CS) | Sec-(SPI) |
| *lp_3141* |  | hypothetical protein lp_3141 |  | 2,98 | Intracellular |  |
| *lp_3160* |  | multidrug transport protein, major facilitator superfamily (MFS) | G: Carbohydrate transport and metabolism | 2,01 | Multi-transmembrane | Sec-(SPI) |
| *lp_3170* | *pmg9* | phosphoglycerate mutase family protein | G: Carbohydrate transport and metabolism | 2,61 | Intracellular |  |
| *lp_3191* | *rrp11* | two component system response regulator | T: Signal transduction mechanisms | 2,03 | Intracellular |  |
|  |  |  | K: Transcription |  |  |  |
| *lp_3243* |  | hypothetical protein lp_3243 |  | 2,41 | Intracellular |  |
| *lp_3250* |  | hypothetical protein lp_3250 |  | 2,29 | Multi-transmembrane | Sec-(SPI) |
| *lp_3305* |  | hypothetical protein lp_3305 |  | 2,19 | Intracellular |  |
| *lp_3318* |  | aldo/keto reductase family protein | R: General function prediction only | -2,13 | Intracellular |  |
| *lp_3353* |  | hypothetical protein lp_3353 |  | 2,18 | Intracellular |  |
| *lp_3421* |  | extracellular protein,gamma-D-glutamate-meso-diaminopimelate muropeptidase | M: Cell wall/membrane/envelope biogenesis | 2,13 | N-terminally anchored (No CS) | Sec-(SPI) |
| *lp_3436* | *glpF5* | glycerol uptake facilitator protein | G: Carbohydrate transport and metabolism | 2,17 | Multi-transmembrane | Sec-(SPI) |
| *lp_3444* |  | Crp family transcriptional regulator | T: Signal transduction mechanisms | 2,04 | Intracellular |  |
| *lp_3472* | *ramP1* | disaccharride transporter, major facilitator super family (MFS) | G: Carbohydrate transport and metabolism | 2,17 | Multi-transmembrane | Sec-(SPI) |
| *lp_3490* |  | FMN-binding protein |  | 2,04 | Intracellular |  |
| *lp_3496* | *ISP2_5* | transposase | L: Replication, recombination and repair | 2,84 | Intracellular |  |
| *lp_3514* | *bglG4* | transcription antiterminator, BlgB family | K: Transcription | 2,18 | Intracellular |  |
| *lp_3540* |  | PTS system, ascorbate-specific EIIC component | S: Function unknown | 2,54 | Multi-transmembrane | Sec-(SPI) |
| *lp_3545* |  | D-arabitol-phosphate dehydrogenase | E: Amino acid transport and metabolism | -2,35 | Intracellular |  |
|  |  |  | R: General function prediction only |  |  |  |
| *lp_3566* |  | N-acetylglucosamine 2-epimerase | G: Carbohydrate transport and metabolism | 2,36 | Intracellular |  |
| *lp_3577* |  | membrane protein | S: Function unknown | 3,53 | Multi-transmembrane | Sec-(SPI) |
| *lp_3579* |  | RNA polymerase (RNAP)-binding regulatory protein, arsenate reductase (ArsC) family, Spx subfamily | P: Inorganic ion transport and metabolism | 2,45 | Intracellular |  |
| *lp_3595* | *rhaB* | rhamnulokinase | G: Carbohydrate transport and metabolism | 2,07 | Intracellular |  |
| *lp_3633* |  | GntR family transcriptional regulator | K: Transcription | -2,31 | Intracellular |  |
| *lp_3650* |  | regulator, Fis family | T: Signal transduction mechanisms | 2,46 | Intracellular |  |
|  |  |  | Q: Secondary metabolites biosynthesis, transport and catabolism |  |  |  |
| *lp_3683* |  | single stranded DNA-binding protein | R: General function prediction only | 2,53 | Intracellular |  |
| *lp_3688a* | *rpmH* | ribonuclease P |  | 2,43 | Intracellular |  |
|  |  |  |  |  |  |  |
| *lp_tRNA08* |  |  |  | 3,05 |  |  |
| *lp_tRNA30* |  |  |  | 2,5 | Intracellular |  |
| *lp_tRNA40* |  |  |  | 20,65 | Intracellular |  |
| *lp_tRNA44* |  |  |  | 6,83 | Multi-transmembrane | Sec-(SPI) |
| *lp_tRNA50* |  |  |  | 19,77 | Intracellular |  |
|  |  |  |  |  |  |  |

^a^ Fold change refers to expression in presence of olive oil 50 % relative to expression in buffer lacking olive oil.

^b^ FDR ≤ 0.0001; p<0.01

^c^ LocateP DataBase (<http://www.cmbi.ru.nl/locatep-db/cgi-bin/locatepdb.py>) CS: CleavageSite; Sec-(SPI): Secretory Pathway I; Sec-(SPII): Secretory Pathway II.
